# Revolutionising portal hypertension diagnosis: the rise of non-invasive techniques in liver cirrhosis

**DOI:** 10.3389/fmed.2025.1647629

**Published:** 2025-08-08

**Authors:** Bocheng Gao, Yumeng Lin, Huimin Zhang, Yulin Li, Shuhua Gou, Peiling Ma, Xueni Zhao, Yue Zhou, Qian Chen, Lan Yuan, Zhongyu Han, Chang Yu

**Affiliations:** ^1^Chengdu University of Traditional Chinese Medicine, Chengdu, China; ^2^School of Medical and Life Sciences, Chengdu University of Traditional Chinese Medicine, Chengdu, China; ^3^Department of Ophthalmology, Nanjing Tongren Hospital, School of Medicine, Southeast University, Nanjing, China; ^4^School of Basic Medical Sciences, Anhui Medical College, Anhui, China; ^5^School of Clinical Medicine, Chengdu University of Traditional Chinese Medicine, Chengdu, China; ^6^School of Pharmacy/School of Modern Chinese Medicine Industry, Chengdu University of Traditional Chinese Medicine, Chengdu, China; ^7^School of Acupuncture and Tuina, Chengdu University of Traditional Chinese Medicine, Chengdu, China

**Keywords:** liver cirrhosis, non-invasive diagnostic techniques, portal hypertension (PH), shear wave elastography (SWE), transient elastography (TE)

## Abstract

Liver cirrhosis is associated with serious complications of portal hypertension (PH), which ultimately causes variceal bleeding and ascites in a life-threatening manner. Non-invasive diagnostic techniques have evolved as an essential tool of early detection and management from a stand point of being dependent on invasive diagnostic techniques. This review summarises the most recent progress in noninvasive diagnostic possibilities in PH in liver cirrhosis in terms of its clinical use and future outlook. A literature review within the last decade and beyond revealed such studies which developed and utilised the indexing technique such as transient elastography, shear wave elastography and other more advanced imaging modalities. Non-invasive techniques which can be used to diagnose PH and monitor it have been made and have been shown to have the possibility of obviating invasive procedures. Given that these are noninvasive techniques, they represent valuable alternatives to invasive PH testing, and future work needs to be directed towards increasing accuracy of these tests and implementing these techniques into routine clinical practise.

## 1 Introduction

Portal hypertension (PH) is a sustained increase in the pressure in the portal venous system which is one of the most common complications of liver cirrhosis (1–3). Increased intrahepatic vascular resistance as a consequence of cirrhosis induced structural remodeling and fibrosis of the liver causes elevated portal venous pressure (4–6). PH is a problem because it is clinically significant as it is associated with a number of severe complications including oesophageal and gastric variceal bleeding, ascites, hepatorenal syndrome and hepatic encephalopathy (7). Variceal bleeding is considered one among the severe complications of PH with the initial bleeding mortality rate ranging up to 30 to 50% and very rarely results into good patients’ outcomes (8, 9). Consequently, early diagnosis and combined therapy for the disease will result in a better prognostic course in patients with liver cirrhosis.

Traditionally, hepatic venous pressure gradient (HVPG) measurement has been considered the “gold standard” for diagnosing PH (10). Portal venous pressure is measured by HVPG, in an accurate reflection as the difference of pressure between the wedged and free hepatic veins (11, 12). However, even though measurement of HVPG is an invasive procedure which has its own risks and complications (bleeding, infection and vascular complication). Furthermore, the measurement of HVPG is a laborious, costly and technically complex procedure that requires use of specialised equipment and trained personnel and thus is not routinely done clinically (13). For this reason, accurate, non-invasive diagnostic tools have been sought in both clinical as well as research areas (14, 15).

In recent years, with the continuous advancement of medical technology, non-invasive diagnostic techniques have gradually become important tools for assessing PH (16). The advantages of non-invasive techniques include their non-invasive nature, repeatability, and lower costs, which reduce patient discomfort and risk (17). Moreover, non-invasive techniques can be used for early screening of PH, monitoring treatment efficacy, and predicting complications. Currently, non-invasive diagnostic techniques mainly include ultrasound-based methods (e.g., transient elastography and shear wave elastography), imaging techniques (e.g., magnetic resonance imaging [MRI] and computed tomography [CT]), and biomarker assays. The continuous development and optimization of these techniques offer new possibilities for the clinical management of PH, especially in resource-limited settings where the application of non-invasive techniques is particularly important (18).

## 2 Non-invasive diagnostic techniques

### 2.1 Ultrasound-based techniques

Doppler ultrasound (DUS) is based on the doppler effect, which is the change in frequency when sound wave is reflected off of moving objects such as red blood cell. A speed and direction of blood flow can be determined by measuring this frequency change (19, 20). These parameters are PVV, HARI, and HHV, to assay hemodynamic changes with PH (21) (Table 1). DUS has a clinical value for the detection of morphological and haemodynamic alterations in patients with advanced liver fibrosis and cirrhosis. Because it is so easily and cheaply available, it is particularly useful for screening and monitoring PH (22).

**Table 1:** Parameters and clinical significance of DUS in the diagnosis of PH.

Parameter	Definition	Clinical significance	Sensitivity/specificity	References
PVV	Blood flow velocity in the portal vein	Reduced velocity indicates PH	Moderate/Moderate	(20)
HARI	Index of blood flow resistance in the hepatic artery	Elevated levels indicate liver fibrosis or cirrhosis	Low/Moderate	(26)
HVV	Doppler waveform of hepatic vein blood flow	Change from triphasic to monophasic waveform indicates PH	High/Moderate	(20)

PH, Portal Hypertension; DUS, Doppler Ultrasound; PVV, Portal Vein Velocity; HARI, Hepatic Artery Resistive Index; HVV, Hepatic Vein Waveform.

However, all of these correlate with liver stiffness and portal pressure and DUS has been shown to correlate with M flap pressures and thrombus location. For example, sheave wave elastography (SWE) measurements of sheave wave elastography (F4) and hepatic vein waveform (tri phasic to monophasic) are highly associated with increase in liver stiffness (23). Nevertheless, sensitivity and specificity of DUS for detecting PH depend on particular parameters (24). Although correlations between PVV and HVV with liver stiffness were strong (25, 26), HARI showed only weak or even no correlation. In general, DUS continues to be a useful tool for the screening and monitoring of PH.

Transient elastography (TE) is a non-invasive technique which can be utilised to measure the liver stiffness by generating and receive low frequency shear waves in hepatic tissue. Shear wave elastography is a technology using a probe to create vibrations in order to determine propagation speed of shear waves through liver tissue, then reflects degree of liver fibrosis and cirrhosis (27). Thus, TE has been widely used for evaluating liver fibrosis and has been validated in different clinical settings to become one of the most popular noninvasive methods for assessment of liver fibrosis (28). We can quantitatively determine liver stiffness measurements (LSM) and have shown high accuracy and reliability of the diagnosis of chronic liver disease, non-alcoholic fatty liver disease (NAFLD) (29) and chronic hepatitis B (30, 31). In addition, early intervention and treatment can be facilitated by TE in detecting liver fibrosis and cirrhosis. An example of that is patients with chronic hepatitis B, where TE can distinguish between inactive chronic carriers and NASH and predict the development of PH (32). However, TE is also provided as a non-invasive and rapid alternative to the cumbersome and risky liver biopsy. However, in some cases, TE results are dependant on factors such as patient obesity, age and diabetes, and therefore additional diagnostic methods may still be necessary (33). TE has also been used extensively in drug development, namely the evaluation of the effects of drugs on liver fibrosis. Changes in LSM before and after treatments can be effectively assessed for efficacy of drugs by monitoring with LSM (34, 35).

Consequently, LSM by the TE have proven very valuable in PH assessment as LSM is well correlated to elevated portal pressure. Studies have shown that liver stiffness values >17 kPa often indicate significant liver fibrosis or cirrhosis and are associated with increased portal pressure (36). This makes TE an important tool for assessing PH, especially in resource-limited settings. However, the accuracy of TE may be limited in moderate fibrosis stages (e.g., F2 and F3) and can be affected by factors such as obesity, inflammation, and cholestasis (37). Therefore, while TE has significant clinical advantages, its limitations should be considered in practice, and other diagnostic tools should be combined to enhance diagnostic accuracy.

SWE overcomes these shortcomings by integrating elastography into conventional B-mode ultrasound (Table 2). Real-time visual guidance allows operators to place a 5 mm sampling box away from large vessels or focal lesions and instantly exclude unreliable measurements, reducing technical failure rates from 7–10% (TE) to <3% (SWE) (27). Deeper penetration and a larger field of view (up to 8 cm) make SWE feasible in obese individuals (BMI > 30 kg m^−2^) and in those with mild ascites. Intrinsic quality metrics—such as propagation maps or quality factors—automatically flag measurements contaminated by respiratory motion, lowering inter-observer variability from 12–15% (TE) to <5% (SWE). SWE is less affected by confounding conditions; in acute hepatitis or cholestasis, SWE-derived liver stiffness rises by <10%, compared with 25–30% for TE, thereby improving specificity for fibrosis. These advances translate into higher diagnostic accuracy: pooled Area Under the Receiver Operating Characteristic curve (AUROC) for ≥F2 fibrosis improves from 0.79 (TE) to 0.87 (2D-SWE), and the failure rate for predicting clinically significant portal hypertension (CSPH) falls from 11% (TE) to 4% (SWE) (38).

**Table 2:** Comprehensive comparison of non-invasive techniques for PH assessment: TE, SWE, and DUS.

Characteristic	TE	SWE	DUS
Technical Principle	Probe-generated shear-wave speed	Real-time shear-wave speed from US push	Spectral/colour Doppler blood-flow analysis
Ease of Use	Dedicated device, moderate complexity	Same US session, minimal extra time	Standard US platform, widely available
Accuracy	Moderate (F4 good; F2-F3 limited)	High (F1–F4 reliable)	Moderate for fibrosis; good for hemodynamic signs of PH
Reproducibility	Moderate	High	Operator- and patient-dependent
Main Clinical Role	Fibrosis staging; PH screening	Fibrosis staging; PH screening & follow-up	PH screening (PVV, HVV), variceal surveillance
Key Limitations	Obesity, inflammation, ascites	Higher cost, vendor variation	Limited sensitivity for early fibrosis

PH, Portal Hypertension; TE, Transient Elastography; SWE, Shear Wave Elastography; DUS, Doppler Ultrasound; PVV, Portal Vein Velocity; HVV, Hepatic Vein Waveform.

Studies supporting the efficacy of SWE for detection and PH of liver fibrosis have been performed. In Zayadeen et al., they showed that SWE was high in the sensitivity and specificity of the liver fibrosis assessment in stage F1–F4 with AUC close to 1 (i.e., high accuracy to assess the liver fibrosis severity) (39). Moreover, the Zhu et al. study found that SWE served well to evaluate the extent of PH of hepatitis B related cirrhosis with significant correlations of liver stiffness to spleen stiffness and HVPG and high accuracy and reliability in diagnosing clinically significant portal hypertension (CSPH) and severe portal hypertension (SPH) (40) (Table 3).

**Table 3:** Summary of clinical validation studies for different elastography techniques in the diagnosis of PH.

Technique	Study type	Key findings	Diagnostic accuracy (%)	Notes	References
TE	Retrospective studies	High correlation between liver stiffness and PH.	80–85%	Effective for advanced fibrosis (F4) but limited in moderate fibrosis stages (F2-F3).	(14)
SWE	Prospective studies	Real-time LSM with high reproducibility.	85–95%	Superior accuracy in detecting early and advanced fibrosis.	(91)
DUS	Prospective studies	Changes in PVV and HVV correlate with PH.	70–80%	Useful for screening but less sensitive to early fibrosis.	(20)

PH, Portal Hypertension; TE, Transient Elastography; SWE, Shear Wave Elastography; DUS, Doppler Ultrasound; PVV, Portal Vein Velocity; HVV, Hepatic Vein Waveform; LSM, liver stiffness measurement.

### 2.2 Advanced imaging techniques

**Figure 1:**
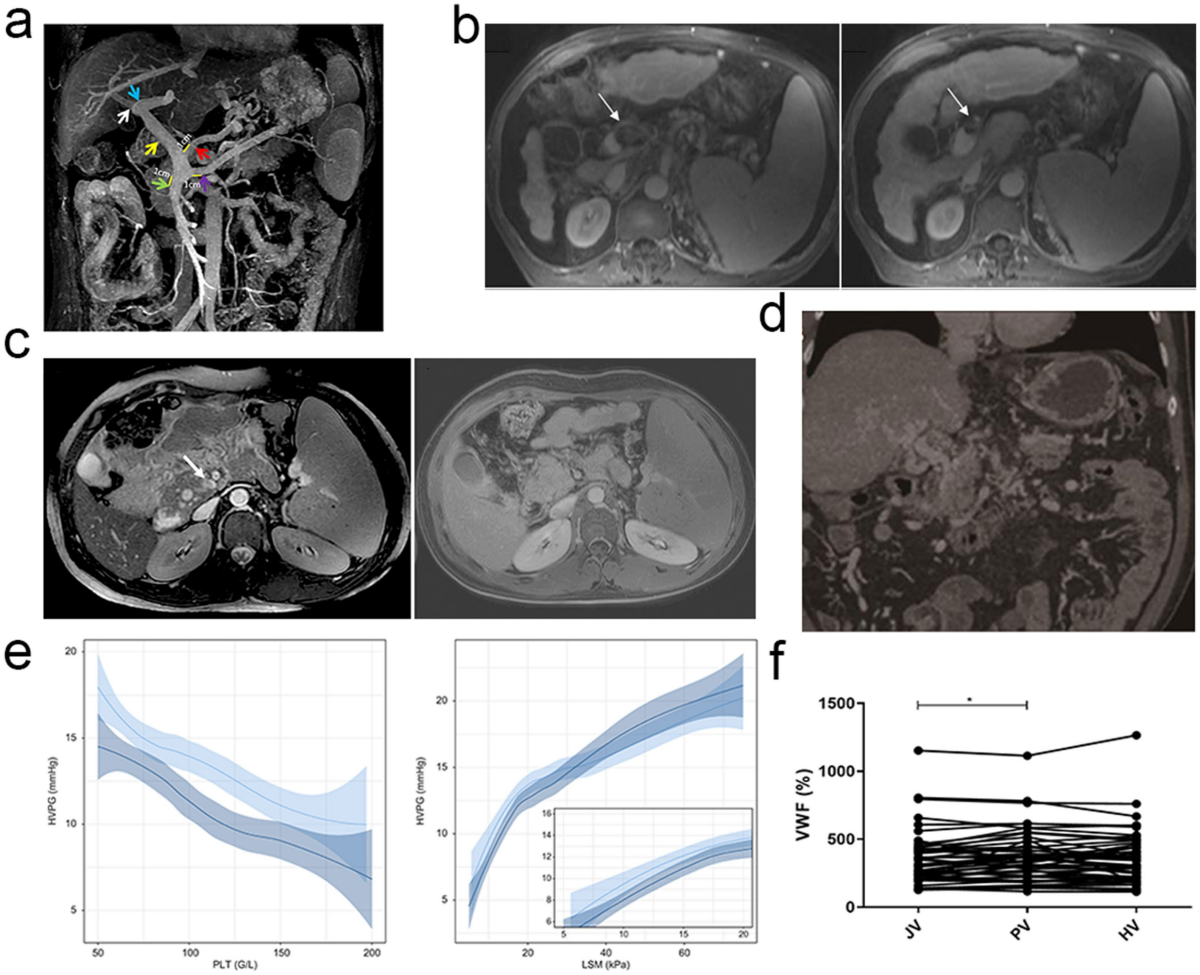
Imaging and Biomarkers in PH. **(a)** MRI clearly visualises the portal vein and its collateral branches, including thrombi and spongy deformities, and measures key diameters for assessing portal pressure. Copyright 2023, Springerlink. **(b)** MRI-detected portal vein thrombosis compared with surgical findings, highlighting the challenges in interpreting small-caliber veins. Copyright 2006, WILEY. **(c)** Contrast-enhanced MRI demonstrating the confluence of the superior mesenteric vein and splenic vein, highlighting enhanced visualisation of hepatic and portal venous structures in a child with EHPVO. Copyright 2021, Springerlink. **(d)** CTA demonstrating sustained recanalization of portal vein thrombosis following transcatheter selective SMA urokinase infusion therapy. Copyright 2017, BPG. **(e)** Negative correlation between platelet counts and HVPG after HCV cure, demonstrating that thrombocytopenia is associated with higher portal pressure, largely due to hypersplenism and increased splenic sequestration. Copyright 2022, ELSEVIER. **(f)** Elevated levels of von Willebrand factor in the portal vein of cirrhotic patients undergoing TIPS placement compared to systemic circulation (jugular vein). This highlights the potential role of VWF as a biomarker in reflecting vascular changes associated with PH in cirrhosis. Copyright 2022, ELSEVIER.

PH has been evaluated with power image which is also a powerful MRI. In view of its capacity to clearly show the opening of the portal vein and its collateral branches as well as the portal body collateral circulation rate with it being highly consistent with that of the arterial portal angiography (41). Moreover, intrahepatic portal veins, their collateral branches, and the thrombi and spongy deformities within them can be clearly visualised by MRI. In addition, it allows assessment of portal pressure on the basis of measurements of liver structure, hepatic and splenic perfusion and splenic artery blood flow (42) (Figure 1a). Whole body macrovascular examinations and pre-shunt surgery or liver transplanting portal vein imaging before surgery can be performed by the advanced MRI methods, e.g., magnetic resonance angiography (MRA) (43) and magnetic resonance portal vein imaging (MRP) (44) (Figure 1b). Furthermore, the low frequency shear wave velocity is an essential diagnostic tool for liver and spleen fibrosis as well as staging and an important basis for quantitative visualisation of the stiffness of the liver and spleen by means of MRI.

Contrast-enhanced MRI (CE-MRI) enhances visualisation of hepatic and portal venous structures and permits better assessment of PH (Figure 1c) (45). It allows the detection of small collateral vessels and the evaluation of liver perfusion by using gadolinium agents. MR elastography (MRE) combines MRI and low-frequency mechanical using to measure the liver stiffness. High sensitivity and specificity of MRE in detecting liver fibrosis and cirrhosis, which are closely related to PH, is demonstrated by MRE. MRE can give quantitative measurements of liver stiffness, and thus a reliable alternative to invasive techniques such as liver biopsy (46).

CT angiography (CTA) is a highly sensitive and specific do which can clearly show vascular structural change in liver and portal venous system. In recent years, with continuation of the development of multilayer spiral CT and dual energy CT technology, CTA diagnosis has become with higher resolution and diagnostic accuracy in diagnosis of liver vascular lesion (47). CTA also can detect atresia or absence of portal vein branches, define portal vein thrombosis and other cirrhosis related complications, investigate formation of collateral circulation (preditcting the risk of rupture and bleeding of oesophageal varices) by injecting contrast agent (48). High resolution CTA reconstruction technology, as reported by recent studies, has ability to more accurately identify the collateral vessels of oesophagogastric varices that have significance for clinical development of individualised treatment plans. Jiang et al. showed the ability of CTA in appraising the treatment efficacy of the transcatheter selective superior mesenteric artery (SMA) urokinase infusion treatment compared to transjugular intrahepatic portosystemic shunt (TIPS) for acute portal vein thrombosis (PVT), and it has unique value (49) (Figure 1d).

CT and MRI are both applied for evaluation of PH, however, have their respective advantages and shortcomings (50). However, MRI provides superior soft tissue contrast and the capability to perform functional imaging (MRE, perfusion) which makes it highly useful for the assessment of liver fibrosis and PH. Secondly, MRI is also a non ionising type of radiation that reduces the radiation exposure to patients. MRI, however, is more expensive and less widely available than CT. Compared to CT, however, CT is faster and more accessible and is therefore appropriate for emergency applications as well as routine imaging. CT is also less sensitive to patient motion and metallic implants. This advantage is however countered by exposure of patients to ionising radiation, which limits its use in the population, especially in young patients or a group of patients requiring frequent follow up imaging (51).

### 2.3 Biomarkers and blood tests

PH has become an important condition to assess, and noninvasive blood tests have come to be an important tool (52). Over the last 10 to 15 years, with a better understanding of pathophysiology of PH, there has been an increased interest in the role of blood markers in predicting and monitoring PH. Non-invasive blood tests that are commonly used include platelet and liver function tests which may give indication of the degree of the disease and the likelihood of PH. Platelet counts are inversely correlated with portal vein pressure and in most cases, thrombocytopenia occurs as a consequence of hypersplensin and increased splenic sequestration (Figure 1e). For example, a prospective study found high sensitivity and specificity (87.9%) for predicting the presence of oesophageal varices when platelet counts were below 144,000/mm^3^, and combined with the assessment of splenic size may further improve diagnostic accuracy (53). In addition, liver function tests—including aspartate aminotransferase (AST), alanine aminotransferase (ALT), and serum albumin—reflect the liver’s synthetic and detoxification capacity and are closely associated with the development of PH. Decreased albumin level is usually associated with hypoproteinemia, which is one of the common complications in patients with PH. Meanwhile, changes in AST and ALT levels not only reflect the degree of hepatocellular damage, but also indirectly indicate the presence of PH (54).

The combined use of multiple biomarkers has significantly improved the predictive accuracy of PH, and vascular hemophilic factor (vWF) and its cleavage products (e.g., vWF-N) in particular have attracted much attention in this field (Figure 1f) (55). vWF is a macromolecular mucin secreted by endothelial cells, and its level is closely associated with PH in cirrhotic patients (56). Studies have shown that elevated vWF levels reflect endothelial cell dysfunction and neovascularization, which are closely related to the pathophysiological mechanisms of PH. vWF elevation is not only associated with the degree of hepatic fibrosis, but also with an increased risk of oesophageal variceal haemorrhage and decompensation in cirrhotic patients. Several studies support the validity of vWF as a diagnostic marker for PH (57). For example, vWF-Ag (vWF antigen) was significantly and positively correlated with HVPG with high sensitivity and specificity. The levels of vWF-Ag in patients with CSPH were significantly higher than normal and can be used as an independent predictor of the severity of PH (58). Diagnostic accuracy can be further improved by combining vWF with additional markers such as platelet count and a disintegrin and metalloproteinase with thrombospondin type 1 motif, member 13 (ADAMTS13) activity (59). For instance, the combination of vWF-Ag and platelet count is a more accurate predictor of the presence as well as the severity of PH. Furthermore, the combined use of vWF-Ag and MELD score can be a better tool to predict the outcomes of cirrhotic patients (60).

Other widely studied composite biomarker models for predicting CSPH include the aspartate aminotransferase-to-platelet ratio index (APRI) and the fibrosis-4 (FIB-4) index (61). These include models that incorporate liver function tests, platelet count, for a global assessment of liver fibrosis and PH risk (62, 63). Nevertheless, in spite of these models, moderate accuracy, the negative predictive value is not very good, indicating a need for more precise biomarker combination.

Recent advancements have introduced novel biomarkers, such as dipeptidyl peptidase-3 (DPP-3) and bile acid metabolites, which have shown potential in further refining the prediction of PH [64; 65]. These biomarkers, when combined with established tests like LSM (e.g., via TE) (64), have demonstrated enhanced diagnostic performance, offering a more nuanced understanding of PH.

## 3 Clinical applications

### 3.1 Screening and early detection

In routine hepatology clinics, the first decision point is whether a patient with cACLD (METAVIR F3–F4) can safely skip screening endoscopy. The Baveno VII consensus offers two evidence-based algorithms. Using the classic rule (transient elastography [TE] < 20 kPa and platelet count > 150 × 10^9^ L^−1^) classifies 60–70% of patients as low-risk for CSPH (HVPG ≥ 10 mmHg), with a pooled negative predictive value of 95% and AUROC 0.84 (Grade A, 16-study meta-analysis, *n* = 2,841) (10). The extended Baveno VII criteria (TE < 25 kPa and platelet > 110 × 10^9^ L^−1^) captures an additional 10% of low-risk patients while maintaining an NPV of 92% (Grade A) (65). In addition to reducing the risk of procedure-related complications, these criteria allow low-risk patients to avoid screening endoscopy while enabling more frequent surveillance and earlier detection of PH-related complications in those who remain at risk (Table 4).

**Table 4:** Application of non-invasive techniques in hepatic cirrhosis screening.

Technique	Detection parameters	Clinical application	Advantages	References
Platelet Count	Platelet levels	Assessing the risk of PH	Non-invasive, rapid, highly repeatable, suitable for initial screening	(65)
Liver Function Tests	ALT, AST, bilirubin, albumin, etc.	Assessing liver function status	Non-invasive, dynamic monitoring, aids in evaluating the severity of cirrhosis	(10)
TE	Liver stiffness	Assessing the degree of hepatic fibrosis and cirrhosis	Non-invasive, easy to operate, highly repeatable	(27)
Ultrasound Elastography	Liver stiffness, portal blood flow	Assessing the degree of hepatic fibrosis and PH	Non-invasive, visual, suitable for primary care hospitals	(27)
MRE	Liver stiffness	Assessing the degree of hepatic fibrosis and cirrhosis	High precision, not affected by BMI, but higher cost	(41)
MRI-PDFF	Liver fat content	Assessing hepatic steatosis and fibrosis risk	High precision, quantitative assessment, but requires high-end equipment	(41)
Biomarker Scoring (e.g., NAFLD Fibrosis Score)	Age, BMI, platelet count, albumin, AAR (AST/ALT ratio), etc.	Predicting the degree of hepatic fibrosis, reducing the need for liver biopsy	Non-invasive, combines multiple indicators to improve diagnostic accuracy	(65)

PH, Portal Hypertension; TE, Transient Elastography; MRE, Magnetic Resonance Elastography; MRI-PDFF, Proton Density Fat Fraction; NAFLD, Non-Alcoholic Fatty Liver Disease; AST, Aspartate Aminotransferase; ALT, Alanine Aminotransferase Ratio.

Obese or diabetic patients with NASH-related cirrhosis represent a rapidly growing cohort. Two large prospective studies (n = 312) have shown that 2-D shear-wave elastography ≥ 9.5 kPa or magnetic resonance elastography ≥ 3.63 kPa identifies CSPH with AUROC 0.88 and 0.90, respectively (Grade B) (66, 67); ongoing multi-centre validation (NCT04832758) aims to confirm these thresholds for formal guideline adoption.

After sustained virological response (SVR), clinicians need to decide whether portal hypertension has regressed sufficiently to withhold further therapy. A 24-week TE follow-up study (*n* = 211) demonstrated that a ≥ 30% decrease from baseline or an absolute value < 12 kPa predicts a ≥ 10% reduction in HVPG with AUROC 0.83 (Grade B) (68), providing an evidence-based trigger to discontinue invasive pressure monitoring.

Acoustic radiation force impulse imaging (ARFI) and 2-D SWE yield AUROC 0.79–0.85 for CSPH in early validation cohorts (69–71), while 3-D hepatic-vein computational-fluid-dynamics modelling has achieved 88% sensitivity and 86% specificity for CSPH using vascular geometry alone (*n* = 94). These techniques are currently confined to tertiary centres with appropriate software and await larger prospective trials before guideline inclusion.

### 3.2 Monitoring and management

**Figure 2:**
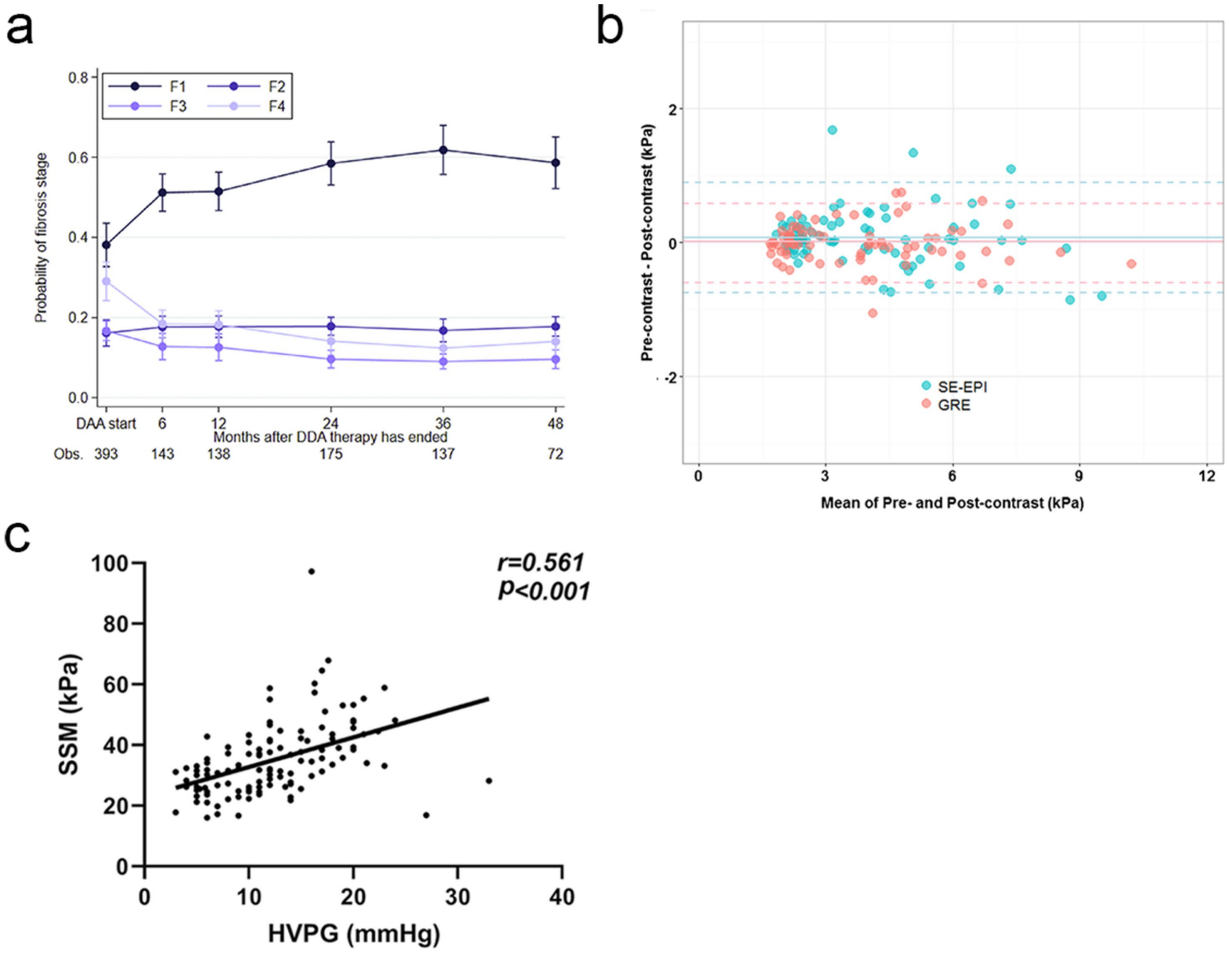
Dynamic Monitoring and Diagnostic Advancements in Liver Fibrosis and PH. **(a)** Over time, the likelihood of fibrosis regression gradually increases, highlighting the dynamic monitoring capabilities of TE. Copyright 2022, ELSEVIER. **(b)** The consistency of liver stiffness (LS) values measured by spin-echo echoplanar imaging-based magnetic resonance elastography (SE-EPI-MRE) and gradient-recalled echo-based MRE (GRE-MRE) is demonstrated. This highlights the technical advancements of SE-EPI-MRE, which enhance diagnostic accuracy through improved signal-to-noise ratio and spatial resolution. Copyright 2017, Springerlink. **(c)** There is a significant positive correlation between SSM and HVPG. Copyright 2024, Springerlink.

Non-invasive techniques play a crucial role in the longitudinal monitoring of PH in cirrhotic patients, mainly including TE, magnetic resonance elastography (MRE) and spleen stiffness measurement (SSM). Of these, TE is one of them, which, because of its convenience and noninvasiveness, is able to frequently monitor the status of the transplanted liver and notify of complications before it becomes a catastrophe, greatly lowering the lifespan. Specifically, TE can be used to assess the trend of postoperative liver fibrosis, providing an important reference for the need of antiviral therapy (Figure 2a) (72). In addition, TE also demonstrates significant advantages in the monitoring of acute cellular rejection (ACR). Through the change of LSM value, TE can reflect the severity of rejection, help early intervention, and buy valuable treatment time for patients (73, 74).

Nevertheless, the clinical impact of TE, MRE and SSM hinges on strict alignment with defined time-windows, patient phenotypes and validated thresholds. In liver-transplant recipients, two such windows dominate. First, from post-operative month 1 to month 12—and every 6 months thereafter—TE is used to screen for fibrotic relapse. Second, within the first 6 months after transplantation, even subtle ALT/AST elevations prompt a TE study to exclude early ACR.

With continuous technological advancements, the imaging sequences of MRE have been significantly optimised. For example, the advent of three-dimensional MRE technology combined with spin-echo echo-planar imaging (SE-EPI) has brought new possibilities. It improves a signal to noise ratio and spatial resolution (Figure 2b). This technology uses a variable flip angle pulse train design, effectively (75). At the same time, the development of new types of equipment has also added new vigour into MRE’s evolution. For example, improved patient comfort provided by the novel devices, such as rectangular, flexible, and soft pneumatic actuators, along with drastic increase of measurement precision has made the MRE much advantageous in the clinical applications (76).

As for Splenic stiffness measurement (SSM), multiple studies have correlated SSM closely with HVPG. For instance, one study demonstrated that the correlation coefficient between SSM at 100 Hz and HVPG was 0.71 (*p* < 0.001), while the correlation coefficient for liver stiffness measurement (LSM) was only 0.17 (*p* = 0.97) (77). That is, SSM could better predict HVPG and is utilised to assess the PH. Another study also found that the strong correlation between SSM and HVPG marked it as a unique contribution to the assessment of PH (Figure 2c) (78). However, it should be noted that when SSM is combined with other non-invasive markers of PH (like platelet count), the risk stratification of PH becomes even more precise and diagnostic uncertainty is lowered. In clinical practice, dynamic monitoring of SSM can also be used to evaluate treatment efficacy and provide a solid basis for individualised treatment plans, thereby assisting clinicians in devising more precise therapeutic strategies for patients.

### 3.3 Integration into clinical guidelines

The integration of non-invasive techniques into clinical guidelines for the management of PH has been a significant advancement in recent years. The Practise Guidance of the American Association for the Study of Liver Disease (AASLD) of 2024 recommends the use of non-invasive examinations to detect CSPH and early NSBBs use when CSPH was identified. Its purpose is to prevent the first decompensation and to decrease the probability of variceal bleeding. The guidelines also highlight the importance of personalised approaches, such as using the “rule of 5” for non-invasive selection of candidates for early NSBB therapy to avoid screening endoscopy (61).

The future directions of clinical guidelines will be towards future validation and refinement of noninvasive tools for the diagnosis of disseminated malignancies in the future. This therefore entails not only cutpoint validation for each of a variety of advanced imaging techniques including MRE and shear wave elastography, but also systematic validation of CSPH estimates from these techniques. Moreover, although most patients with portal hypertension have an excellent clinical prognosis, there is worth in looking for non-invasive methods to monitoring changes in HVPG and identifying conditions under which clinical recompensation can occur, and thereby permit use of strategies for de-escalation of HVPG monitoring and therapy in patients with portal hypertension.

Thus, PH is now of great interest in terms of application of personalised medicine in PH. With the combination of advanced biomarker and imaging based on non-invasive techniques we are now able to undertake customised treatment of an individual’s risk profile. To predict the risk of CSPH, the ANTICIPATE-CSPH model had been developed using noninvasive predictors. Ongoing efforts are being made to refine these models for specific patient populations, such as those with NASH (17).

Moreover, personalised approaches may involve the use of novel pharmacological agents, such as statins, which have shown promise in reducing HVPG and improving survival in patients with cirrhosis. Future research will focus on confirming the safety and efficacy of these agents, particularly in combination with existing therapies like NSBBs (79, 80) (Table 5).

**Table 5:** Application and future directions of non-invasive techniques in the management of PH.

Theme	Content	Detailed description
Current Status of Non-Invasive Techniques	Integration of Techniques	The 2024 AASLD guidelines recommend using non-invasive assessments to identify CSPH. These techniques include: 1. “5-Rule”: A non-invasive parameter-based assessment method for screening candidates for early NSBB therapy, avoiding unnecessary screening endoscopy. 2. Biomarkers Combined with Imaging: By combining blood biomarkers (such as liver function indicators, inflammatory markers) and imaging techniques (such as liver ultrasound, CT), the presence and severity of PH are preliminarily assessed.
	Clinical Application	The main goals of non-invasive techniques are: 1. Early Identification of CSPH: To identify high-risk patients as early as possible through non-invasive means, so as to initiate preventive treatment (such as NSBBs) in time and reduce the risk of variceal bleeding and decompensation. 2. Reducing the Use of Invasive Examinations: To avoid unnecessary endoscopy, reduce patient suffering and medical costs, and at the same time, reduce the risk of complications that may be caused by invasive examinations.
Future Validation Directions	Technique Validation and Optimization	Future guidelines will focus on the systematic validation of non-invasive diagnostic tools, including: 1. MRE: By assessing liver stiffness to indirectly estimate portal pressure. Future research will focus on determining the diagnostic cut-off points of MRE to more accurately identify CSPH. 2. SWE: An ultrasound imaging technique that can measure liver stiffness in real-time. Research will further optimise the parameter settings and diagnostic thresholds of SWE to enhance its clinical application value.
	Monitoring and Decompensation Assessment	Research will focus on developing non-invasive methods to monitor changes in HVPG, with the goals of: 1. Confirming the Threshold for Clinical Decompensation: Using non-invasive means to determine the critical values of HVPG changes, so as to adjust treatment plans in a timely manner. 2. Reducing Monitoring Intensity: Avoiding frequent invasive HVPG measurements to reduce patient burden and medical costs.
	Precision Assessment and Treatment	Non-invasive techniques combined with biomarkers and imaging provide a basis for personalised medicine, including: 1. Risk Prediction Models: Such as the ANTICIPATE-CSPH model, which uses non-invasive parameters to predict the risk of CSPH occurrence and provides early intervention for high-risk patients. 2. Personalised Treatment Plans: Formulating individualised treatment strategies based on the patient’s liver disease aetiology (such as non-alcoholic fatty hepatitis), liver function status, and the degree of PH.
Potential for Personalised Medicine	Application of Novel Drugs	Research is exploring the potential of novel drugs (such as statins) in reducing HVPG and improving survival rates in patients with cirrhosis. Future research will focus on: 1. Safety and Efficacy of Drugs: Conducting clinical trials to verify the safety and efficacy of novel drugs in different patient populations. 2. Combined Therapy Strategies: Investigating the combined application of novel drugs with existing treatments (such as NSBBs) to explore their synergistic effects in reducing PH and improving prognosis.

PH, Portal Hypertension; CSPH, clinically significant portal hypertension; NSBB, non-selective beta-blocker; MRE, Magnetic Resonance Elastography; SWE, Shear Wave Elastography; HVPG, hepatic venous pressure gradient; AASLD, American Association for the Study of Liver Disease.

## 4 Challenges and future directions

### 4.1 Technical limitations

Despite significant advancements in non-invasive diagnostic techniques for PH, challenges remain regarding accuracy and reproducibility. Obesity and ascites are critical factors affecting the accuracy of ultrasound elastography (81). In obese patients, the higher attenuation of low-frequency shear waves by adipose tissue leads to a higher rate of failed measurements. For instance, FibroScan has a failure rate of up to 22–25% in patients with a BMI greater than 30 kg/m^2^ (82). Ascites can further interfere with the propagation of ultrasound waves, reducing the reliability of measurements.

For example, these results depend on inconsistency in operator skill and experience. This has been shown to be influenced by subjective perceiver perception, the probe pressure, and region of interest (ROI) selection, but measures the quantities of interest. For example, experience of the operator and measurement situation may generally greatly condition TE and SWE reproduction. Moderation fibrosis (F2–F3) may perform ultrasound elastography at the lower level of sensitivity and specificity. For example, TE is 76% sensitive and 88% specific in the diagnosis of moderate fibrosis (83). Although SWE has proved to be very accurate for measurement of liver fibrosis, hepatic congestion and cholestasis can still influence SWE measurements (84).

Other variables that can also influence the results of ultrasound elastography include the degree of hepatic inflammation, alcohol consumption and extrahepatic factors, for example, heart failure (85, 86). However, ongoing research is placed on these limitations in these techniques and protocols to substantiate this need for refinement of techniques and protocols to improve the reliability and accuracy of these noninvasive assessments across a variety of clinical settings (87).

### 4.2 Clinical validation

Large scale multicenter studies are needed to fully establish the clinical application value of non-invasive diagnostic techniques in PH. Such studies can yield reliable data for the diagnostic accuracy, sensitivity and specificity of various noninvasive methods and facilitate their general propagation (77, 88).

While the clinical application of these techniques appears to be feasible for some, further validation of their use is still needed to prove diagnostic potential from noninvasive data available from existing data. For instance, MRE and dynamic contrast-enhanced MRE (DCE-MRE) have shown promise in initial studies (89), but their clinical utility needs to be validated in larger cohorts. Furthermore, although MRE has had success for evaluating liver fibrosis and disease (90), the inherent high cost and high hardware requirements of MRE prevent its widespread use in clinical practise.

To be more generalizable with respect to health care systems and patient populations, multicenter studies become important. However, comparison with other (particularly noninvasive) techniques is needed to validate the application. However, correlation of liver stiffness and spleen stiffness with PH has been demonstrated but there are multicentre studies yet to be validated for prediction by these indicators (91, 92).

This also leads to current research in which non-invasive diagnostic techniques cannot yet be safely applied in specific populations. In certain populations with human immunodeficiency virus (HIV) / hepatitis C virus (HCV) coinfection, non-alcoholic fatty liver disease or alcoholic cirrhosis, the effectiveness of non-invasive techniques may vary (93, 94). For these techniques to be considered useful and effective on a multicenter basis, the patient populations need to have varying etiologies, ethnicities, and disease stages.

### 4.3 Emerging technologies

Ideas of artificial intelligence (AI) and machine learning (ML) have improved the noninvasive diagnosis of PH significantly (95), as the AI algorithms can scan imaging exams and blood tests to detect very small changes, which a human may not realise. Therefore, deep convolutional neural networks (CNNs) have offered much potential for recognition of CSPH patients on CT or MRI images with greater accuracy, in comparison with the conventional diagnostic methods (96).

Furthermore, with some relatively recent studies, AI has also been used to analyse CSPH patients’ liver vascular reorganisation, and it was found that CSPH patients have some unique redistribution of liver vascular (97). Based on these findings, new state of the art models were built, and the AI models trained reached and exceeded the standard of care diagnostic performance. Moreover, machine learning models help increase diagnostic accuracy by their capacity to differentiate minimal differences in measurements of liver stiffness or blood biomarkers that are linked with PH (98, 99) (Table 6).

**Table 6:** Known biomarkers for portal hypertension (PH)—summary of clinical relevance.

Biomarker / test	Normal range	Clinical relevance & cut-off for PH	References
Liver stiffness (TE)	<7 kPa	>17 kPa → high probability of CSPH (HVPG ≥10 mmHg)	(10, 36)
Liver stiffness (SWE)	<7 kPa	≥9.5 kPa → CSPH in NASH cirrhosis (AUROC 0.88)	(66, 67)
Spleen stiffness (SSM, 100 Hz)	<18 kPa	≥46.8 kPa → CSPH (r = 0.71 vs. HVPG)	(77, 78)
Platelet count	150–400 × 10^9^ L^−1^	<110 × 10^9^ L^−1^ → high PH risk (Baveno VII extended criteria)	(10, 65)
APRI	<0.5	>1.0 → advanced fibrosis & PH risk	(61, 62)
FIB-4 index	<1.30 (age <65 yr)	>2.67 → advanced fibrosis & PH risk	(61, 63)
vWF-Ag	50–160%	>200% → CSPH (independent predictor, AUROC 0.83)	(56, 58)
ADAMTS13 activity	≥50%	<30% plus vWF↑ → higher bleeding/decompensation risk	(59)
AST/ALT ratio (AAR)	<1	>1 → fibrosis progression & PH risk	(61)
Albumin	35–50 g L^−1^	<35 g L^−1^ → decompensation risk, correlates with PH severity	(54)
Bile acid profile	Total <10 μmol L^−1^	Elevated conjugated bile acids → portal pressure ↑	(102)
DPP-3	<130 ng mL^−1^	↑ levels correlate with HVPG and circulatory dysfunction	(103)

PH, Portal Hypertension; TE, Transient Elastography; kPa, kilopascal; CSPH, Clinically Significant Portal Hypertension; HVPG, Hepatic Venous Pressure Gradient; SWE, Shear Wave Elastography; NASH, Non-Alcoholic Steatohepatitis; SSM, Spleen Stiffness Measurement; Hz, Hertz; LSM, Liver Stiffness Measurement; PV, Portal Vein; PLT, Platelet count; APRI, AST-to-Platelet Ratio Index; FIB-4, Fibrosis-4 Index; vWF-Ag, von Willebrand Factor Antigen; ADAMTS13, A Disintegrin And Metalloproteinase with ThromboSpondin type 1 motif, member 13; AST, Aspartate Aminotransferase; ALT, Alanine Aminotransferase; AAR, AST/ALT Ratio; g L^−1^, grams per litre (albumin units); μmol L^−1^, micromoles per litre (bile acid units); DPP-3, Dipeptidyl Peptidase-3; ng mL^−1^, nanograms per millilitre (DPP-3 units).

While these technologies are increasingly used, incorporating data standardisation, algorithm transparency, and all types of ethical concerns, in particular, those related to data protection and data security, remains to be solved. Future research on shaking off these challenges is necessary to realise the extended potential of AI and ML in the noninvasively diagnosis of PH (100, 101).

## 5 Conclusion

Finally, significant and potential advances in clinical application have already been achieved for the noninvasive techniques for PH diagnosis. TE, SWE, Doppler ultrasound and with sophisticated imaging tools (MR, CT) have been utilised circumstantially with promising accuracy and reproducibility in detecting and monitoring PH. In addition, these non-invasive methods have become much more powerful in terms of diagnostic using biomarkers such as von Willebrand factor. Because of that and because they represent valuable alternatives to invasive procedures that cause patient discomfort and complications, they are also typically used.

They have the potential of building a revolution in clinical practise, in patient care and in resource utilisation as non-invasive techniques. These methods would allow for early detection and longitudinal PH monitoring and earlier interventions to promote patients’ outcomes and prevent complications, such as variceal bleeding and ascites, which are an outcome of PH. In addition, clinical workflows are facilitated by using non-intrusive techniques that allow reduction in invasive procedures such as HVPG measurement and hence better allocation and a better resource efficiency of healthcare. Additionally, they showcase the clinical benefits of the advancements via personalisation of the treatment plans that are thus guided by individual patient profiles.

However, there are still many ways to make progress and the clinical validation in these extensions can be justified. Since validation of such non-invasive techniques necessarily must be large scale, multicentre and cover a clinically relevant range of patient population, the parameters of sensitivity, specificity, and the positive and negative predictive values are the necessary ones to provide. The widespread use will benefit from standardisation of protocols and harmonisation of diagnostic platforms in order to increase consistency. Furthermore, the diagnostic precision is augmented and the diagnostic model is integrated using such emerging technologies such as the artificial intelligence, respectively machine learning. In addition, future research should focus on evaluation of long term effect on patient outcomes and health care costs to enabling this method to be embedded in the clinical guidelines.

Notwithstanding these encouraging perspectives, several practical limitations remain. Elastography (TE/SWE) can yield unreliable results in patients with ascites, obesity, or acute inflammation, resulting in non-diagnostic studies in roughly 10–15% of cases. MR- and CT-based pressure estimations are highly sensitive to acquisition protocols and post-processing choices, leading to vendor- and centre-specific cut-offs that hinder universal applicability. Biomarkers such as von Willebrand factor, while additive, lack liver specificity and may be confounded by systemic endothelial dysfunction in heart failure or sepsis, thereby lowering specificity. Lastly, most existing validation cohorts are small or single-centre; multicentre studies report broader confidence intervals for sensitivity and specificity, emphasising the need for larger, harmonised datasets before routine clinical adoption can be recommended.
